# The Developments and Iterations of a Mobile Technology-Based Fall Risk Health Application

**DOI:** 10.3389/fdgth.2022.828686

**Published:** 2022-04-27

**Authors:** Katherine L. Hsieh, Mikaela L. Frechette, Jason Fanning, Lingjun Chen, Aileen Griffin, Jacob J. Sosnoff

**Affiliations:** ^1^Department of Internal Medicine, Section on Gerontology and Geriatric Medicine, Wake Forest School of Medicine, Winston-Salem, NC, United States; ^2^Department of Kinesiology and Community Health, University of Illinois at Urbana-Champaign, Champaign, IL, United States; ^3^Department of Health and Exercise Science, Wake Forest University, Winston-Salem, NC, United States; ^4^Department of Physical Therapy, Rehabilitation Science, and Athletic Training, University of Kansas Medical Center, Kansas City, KS, United States

**Keywords:** mHealth, smartphone, older adults, multiple sclerosis, non-ambulatory, fall prevention

## Abstract

Falls are a prevalent and serious health concern across clinical populations. A critical step in falls prevention is identifying modifiable risk factors, but due to time constraints and equipment costs, fall risk screening is rarely performed. Mobile technology offers an innovative approach to provide personalized fall risk screening for clinical populations. To inform future development, this manuscript discusses the development and testing of mobile health fall risk applications for three unique clinical populations [older adults, individuals with Multiple Sclerosis (MS), and wheeled-device users]. We focus on key lessons learned and future directions to improve the field of fall risk mHealth. During the development phase, we first identified fall risk factors specific to each population that are measurable with mobile technology. Second, we determined whether inertial measurement units within smartphones can measure postural control within the target population. Last, we developed the interface of each app with a user-centered design approach with usability testing through iterative semi-structured interviews. We then tested our apps in real-world settings. Our cumulative work demonstrates that mobile technology can be leveraged to provide personalized fall risk screening for different clinical populations. Fall risk apps should be designed and tailored for the targeted group to enhance usefulness and feasibility. In addition, fall risk factors measured with mobile technology should include those that are specific to the population, are measurable with mobile technology, and can accurately measure fall risk. Future work should improve fall risk algorithms and implement mobile technology into fall prevention programs.

## Introduction

Approximately one in four older adults fall each year (1). Falls can result in serious health consequences, including fractures (2), traumatic brain injuries (3), and even death (4). Falls also result in activity curtailment, isolation, and a loss of independence (5, 6). Because of these devastating consequences, preventing falls is critical to maintaining independence and a high quality of life for older adults.

Over the last decade, hundreds of clinical interventions have been implemented to investigate fall prevention strategies (7, 8). Over 159,000 older adults have participated in these clinical trials to investigate the effect of exercise, vitamin D, dual-task training, and/or education on falls (9). Ultimately, these interventions have determined that falls are a complex and multifactorial problem, and current approaches have limited success. Despite the increase in the knowledge base concerning fall risk and prevention strategies, the age-adjusted fall death rate among older adults has nearly doubled in the last decade (10). There is a great need for innovative approaches to prevent falls.

A critical step in falls prevention is identifying modifiable risk factors. The American Geriatric society as well as the Centers for Disease Control and Prevention (CDC) recommends screening of fall risk for older adults at least annually by physicians (11, 12). However, due to time constraints and expensive equipment, fall risk screening is rarely performed clinically (13). Mobile technology offers an alternative approach to measure multiple fall risk factors in remote-based settings. Inertial measurement units embedded in smartphones allow for precise measurement of balance and gait (14), while websites and surveys can allow for the collection of patient-reported risk factors such as medication use, health history, and fear of falling. Moreover, mobile technology is portable, affordable, and ubiquitous, and with the disruption of in-person preventative health care during the COVID-19 pandemic, mobile technology offers promise for remote-based fall risk screening.

Over the past 5 years, our research group has developed and tested several iterations of a fall risk mobile health application (app), Steady^TM^. The purpose of Steady is to provide users with accessible, objective, and personalized fall risk screening by measuring modifiable fall risk factors specific to older adults (15). However, other clinical populations who are also at high risk for falls have modifiable risk factors that differ from that of older adults. Therefore, leveraging mobile technology for other clinical populations requires developing unique algorithms and design approaches. The purpose of this paper is to discuss the development and testing of a mobile health fall risk application for older adults, individuals with Multiple Sclerosis (MS), and wheeled-device users. We discuss key lessons learned in mobile health fall risk screening in each population and future directions to prevent falls across clinical populations.

## Fall Risk mHealth Development

**Figure 1:**
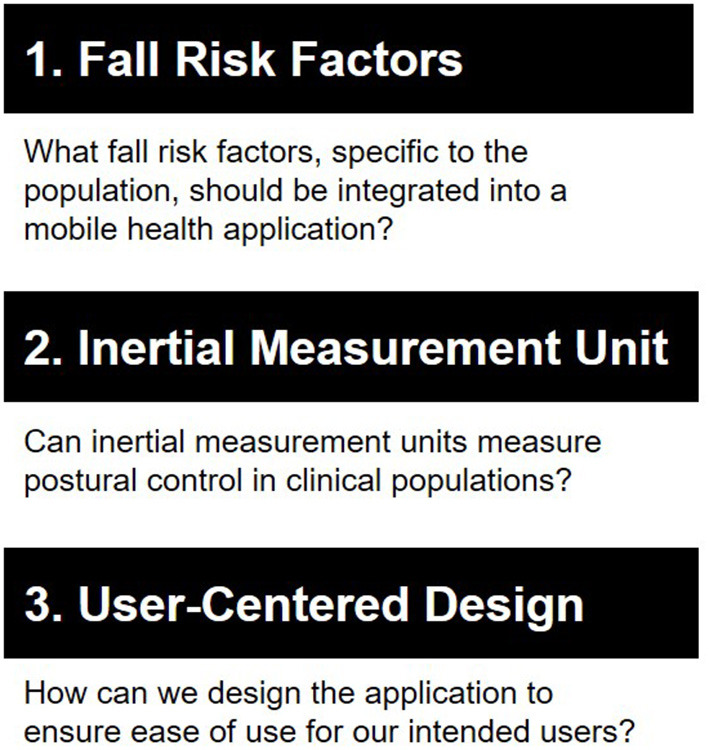
We followed a three-step approach to develop each fall risk application. First, we identified fall risk factors specific to each population that can be measured using mobile technology. Second, we determined whether inertial measurement units within smartphones can measure postural control. Last, we designed the application with a user-centered approach.

Developing a fall risk mHealth application involves three key phases, as depicted in Figure 1. The first step is to identify fall risk factors specific to the target population that can be measured with mobile technology. Because falls stem from a complex interplay of risk factors, it is important to review the literature and identify key risk factors that can be measured with mobile technology (16). Postural instability is often a key risk factor across multiple populations (17–19), and therefore, the second step in mHealth development is to determine if inertial measurement units embedded within smartphones can measure postural control. Postural control is commonly measured with force plates or pressure walkways. While these can provide precise measures of postural control, they are expensive and limited to laboratory settings. Accelerometers can detect changes in mobility that are more sensitive than clinical assessments (14). Therefore, it is important to test if smartphone accelerometers are comparable to research-grade equipment in measuring postural control. The third step is to integrate the fall risk factors identified into a mHealth app, undergoing a user-centered design process. This process focuses on ensuring that the end product will be user-friendly for the intended users.

This multi-phase approach was adopted for the development of three mHealth fall risk assessment apps designed for different clinical populations. The first app, Steady, was developed for assessing fall risk in community-dwelling older adults. Steady-MS was developed for individuals living with multiple sclerosis. Lastly, Steady-Wheels was developed for wheeled device users, defined as individuals who utilize wheeled mobility assistive devices for 85% of their mobility needs (20). Importantly, although each of these clinical populations is at heightened risk of falls, there was a high likelihood of different risk factors and use needs.

The first step in developing a fall risk application was to identify key risk factors measurable with mobile technology. Research over the past decade has demonstrated that falls stem from an interplay of multiple risk factors (21). Therefore, identifying the most relevant risk factors specific to a target population is critical for an accurate measure of fall risk. Thorough literature searches were conducted to identify all possible fall risk factors. These options were then synthesized to include those that can be implemented into each mHealth app. For older adults, key fall risk factors included age, sex, fall history, balance completion, and balance confidence. For people with MS, key fall risk factors included age, fall history, walking ability, postural control, and MS disability. For wheeled device users, this included seated postural control, fall history, and fear of falling. These measures were specifically selected because they can be easily implemented into a mHealth interface while providing valid measures of fall risk.

Because postural instability was a fall risk factor common across all three populations, the next step was to determine whether smartphone accelerometry can measure standing and/or seated postural control. For older adults, we compared smartphone accelerometry to a force plate in 30 older adults while they performed progressively challenging balance tasks (22). Smartphone accelerometry was comparable to a force plate in measuring root mean acceleration in the mediolateral (ρ = 0.44–0.66) and anteroposterior directions (ρ = 0.42–62). In addition, root mean acceleration in the anteroposterior (area under curve = 0.76–0.84) and vertical directions (area under curve = 0.75–0.77) discriminated between older adults at high and low fall risk (22).

In those with MS, there were similar results that smartphone accelerometry is comparable to a research-grade accelerometer in measuring postural control (23). In 27 individuals with MS, there were moderate to strong correlations between root mean square acceleration in the mediolateral (ρ = 0.88–1.0) and anteroposterior (ρ = 0.93–0.99) directions and 95% confidence area acceleration (ρ = 0.92–0.99) between a smartphone and research-grade accelerometer (Opal sensor, ADMP, Inc.). In addition, root mean square acceleration (area under curve = 0.70–0.92) and 95% confidence ellipse acceleration (area under curve = 0.85–0.89) could discriminate between assisted device users and non-assisted device users.

In wheeled-device users, we found that smartphone accelerometers can also measure seated postural control. In 11 wheeled-device users performing seated balance tasks, there were moderate to strong correlations between smartphone accelerometry and a research-grade accelerometer (Opal sensor, ADMP, Inc.) for maximum acceleration (ρ = 0.76–0.98), root mean square acceleration (ρ = 0.92–0.98), and 95% confidence area ellipse (ρ = 0.72–0.98). There was also moderate reliability with smartphone accelerometry, with the strongest reliability during the eyes closed task (ICC = −0.10 to 0.98). Furthermore, mediolateral root mean square acceleration was better able to discriminate between those with and without impaired postural control (area under curve = 0.43–0.90).

The last step was to develop a mHealth interface incorporating these fall risk measures. During the development of our apps, we followed a user-centered approach, designing the app specifically for our end-users in mind. For instance, for older adults, we considered visual and hearing impairments that are associated with aging (15). For people with MS, we also considered cognitive impairments and loss of manual dexterity that are symptoms of MS (24), and for wheeled device users, we considered cognitive load, dexterity, and sensory function. After designing the initial app, we performed semi-structured interviews with our intended users to determine likes, dislikes, and how to improve each app. We also asked participants how they preferred to view their fall risk score, such as through a graph, chart, or scale. We modified each app based on participants' feedback and performed more interviews until there were few usability concerns. This ultimately resulted in a usable and useful fall risk app for older adults, people with MS, and wheeled device users.

## Fall Risk mHealth Testing

Following the iterative process of developing a usable fall risk app, we tested the app in real-world settings to determine the feasibility of individuals measuring their own fall risk. In older adults, we recruited 15 older adults in a retirement center to use independently use Steady and understand their fall risk score (25). Participants also completed clinical fall risk assessments, such as the Timed Up and Go, Berg Balance Scale, and Physiological Profile Assessment, and returned in a week to repeat all tasks. We found that the Steady algorithm had a moderate to strong correlations with the Physiological Profile Assessment (ρ = −0.65; *p* = 0.009), Activities Balance Confidence Scale (ρ = 0.70; *p* = 0.004), Berg Balance Scale (ρ = 0.88; *p* < 0.001), and Timed up and Go (ρ = −0.080; *p* < 0.001). In addition, there was high test-retest reliability (ICC = 0.90; *p* = 0.001). This suggests that Steady is comparable to clinical fall risk assessments and reliable over time. In addition, older adults were able to safely use Steady in a retirement home with support from research staff.

In people with MS, our research staff met with participants virtually over video and watched as 13 participants completed Steady-MS independently in their own homes. They also completed questionnaires online including the Falls Efficacy Scale, MS Walking Scale, and Patient Determined Disease Steps. Participants were safe using the app and had little to no issues navigating through the app. Moreover, there were moderate to strong correlations between the Steady-MS algorithm and the Patient Determined Disease Steps (ρ = 0.65; *p* = 0.02), MS Walking Scale (ρ = 0.70; *p* = 0.01), and Falls Efficacy Scale (ρ = 0.88; *p* < 0.001). These results offer the potential for safe and feasible use of Steady-MS in remote-based settings.

This work implementing Steady and Steady-MS in real-world environments suggests that older adults and people with MS can potentially use a fall risk app to measure their risk for falls. This offers the opportunity to integrate mobile fall risk with telemedicine or community-based exercise programs to include fall risk screening as a step in the healthcare system.

## Lessons Learned

When developing and testing each fall risk app, we identified important lessons learned to facilitate the future fall risk mHealth apps. In the development phase, we identified five lessons. First, we had an interdisciplinary team who had a diverse set of skills to help create Steady, Steady-MS, and Steady-Wheels. This team consisted of biomechanists, an app developer, behavioral scientist, and human factors engineer. Each member of this team offered unique skills to contribute to the fall risk app, and we recommend having an interdisciplinary team when developing health technology.

Second, when measuring postural control, we decided to have participants hold the phone against their chest as a proxy of center of mass. We chose this location as it is more feasible for participants to use on their own rather than the lower back. For older adults, those with MS, and wheeled device users, we found that the chest was a suitable location. This allowed participants to hold the phone with one hand against their chest and use the second hand for postural support if needed. During the development of Steady, we also tested postural control with tablets but found that older adults had difficulty holding a larger tablet against their chest. Therefore, moving forward we used a smartphone device.

Third, we picked specific design considerations for our intended users. For instance, visual impairments are commonly associated with aging and MS. Therefore, for all apps, we picked large font sizes and contrasting colors. Cognitive impairment is also common among older adults and people with MS. Therefore, we kept instructions simple with demonstrative graphics and kept questionnaires short to prevent cognitive overload. By integrating these design features up front, we prevented participants from expressing these as usability concerns, reducing the number of iterations during the testing phase. In addition, during usability testing, we also came across concerns that were unexpected during the design phase. For instance, during the balance tasks, we noticed that some participants read only the instructions, while others only looked at graphics. Because different participants have different learning strategies, we learned it was important to integrate both clear written and visual instructions.

Fourth, it is important to ensure that participants are safe. Falls can result in serious injury (4), and we aimed to prevent users from potentially falling when using our apps. We included instructions before the balance tasks to stand near a sturdy object, such as a wall or table. For wheeled-device users, we included instructions to power off their electric wheelchair or to lock their wheels. For each balance task, we also included the option to skip any tasks that participants felt unsafe performing. Because users may be at high fall risk, it is important to include safety instructions and options to ensure safety when using the fall risk app.

Last, we found that a clear graphic, such as a color-coded scale, to depict an individual's fall risk score was useful to help participants understand their fall risk. If participants cannot correctly interpret their score, then a fall risk app has limited benefit. During semi-structured interviews, we asked participants how they would like to receive their score, and provided examples of graphs and images. Across populations, we found general agreement in using colors (i.e., red for bad, green for good), along with numbers and words (i.e., low, moderate, high). We recommend following a similar format for future fall risk apps.

When testing Steady and Steady-MS in real-world settings, we also learned two valuable lessons to implement fall risk mHealth apps. First, participants were successful in independently measuring their fall risk. However, they were interested in understanding the next steps to reduce their risk of future falls. This opens the opportunity to provide fall prevention resources in a future iteration, or to integrate a fall risk app with health care providers who can provide fall prevention strategies. Second, it is important to continue to iterate and refine mHealth apps. Some participants reported wanting to track their changes in fall risk to determine if they improve. A future iteration could include a tracker or additional features that participants find useful. While the development and testing phase includes multiple iterations, there is continuous room for improvement to reiterate and refine fall risk apps.

## Discussion

**Figure 2:**
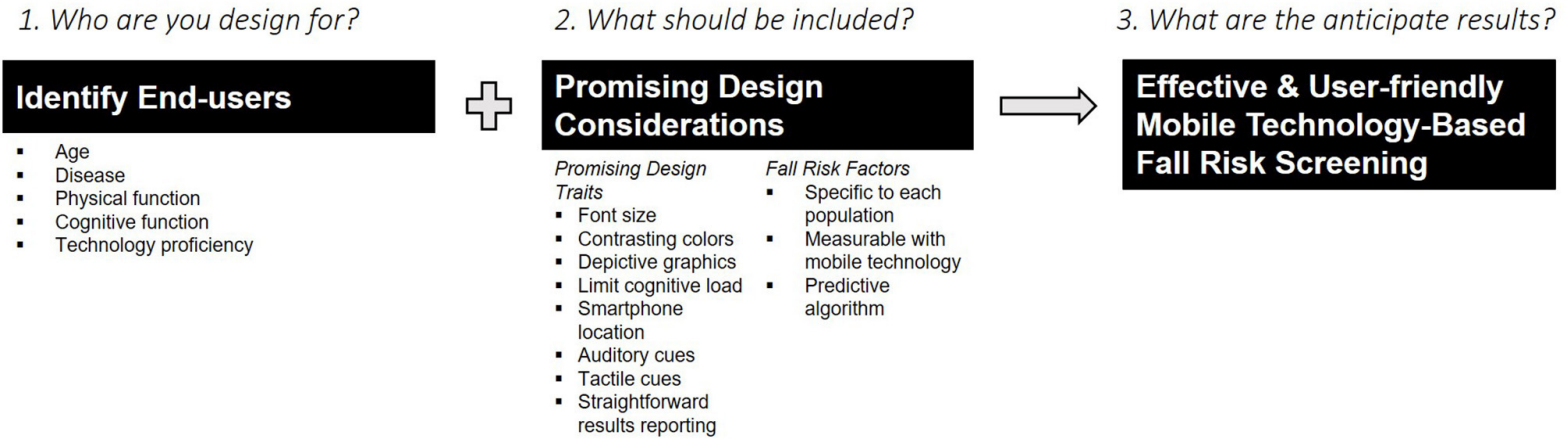
Developing a successful, effective, and user-friendly fall risk application involves identifying who the end-users are and including promising design considerations.

The purpose of this review was to discuss the development and testing of smartphone-based fall risk screening in clinical populations, a critical step in preventing falls. We developed fall risk apps for older adults, people with MS, and wheeled device users, populations that are at high risk for falls. Because of each group's unique fall risk factors, we designed and tested each app for the targeted population. Our results indicate that different clinical populations require tailored fall risk screening and that smartphone-based screening offers the potential to provide personalized screening. Additionally, when developing fall risk apps for future use, we learned important design features to consider and modifiable risk factors to include (Figure 2).

A key step in falls prevention is identifying modifiable risk factors and intervening upon those risk factors. Our results suggest that modifiable risk factors can be measured with smartphone technology through accelerometers and self-reported questionnaires. Moreover, different populations have unique modifiable risk factors, and these can be personalized with smartphone technology. Table 1 summarizes differences in key features for each fall risk application. For instance, different balance tasks were chosen to challenge postural control for older adults, those with MS, or wheeled device users. Different algorithms were also developed for each app to include risk factors specific to each population. Design features, too, had similarities when initially designed for each population, but had differences after feedback from individual interviews. Overall, while smartphone technology is a unique tool that can increase access to fall risk screening, mHealth apps need to be tailored toward the targeted users.

**Table 1:** Similarities and differences between Steady, Steady-MS, and Steady-Wheels.

	**Steady**	**Steady-MS**	**Steady-Wheels**
Target population	Older adults	Individuals with multiple sclerosis	Individuals who use wheeled mobility devices
Fall risk factors	• Age • Balance completion • Fear of falling • Fall history	• Postural control • Perceived walking ability • Balance confidence • Fall history	• Seated postural control • Fear of falling • Fall history
Design considerations	• Visual impairment • Hearing impairment • Cognitive impairment	• Visual impairment • Manual dexterity • Cognitive impairment	• Visual impairment • Manual dexterity • Cognitive impairment • Non-ambulatory
Design solutions	• Larger font sizes • Contrasting colors • Audio cues • Balance tasks: eyes closed, single-leg, sit-to-stand	• Larger font sizes • Contrasting colors • Visuals to depict instructions • Balance tasks: eyes closed, tandem, single leg	• Larger font sizes • Brief instructions immediately preceding a task • Selection options and buttons were made large • Typing responses avoided • Balance tasks: static sitting with eyes open, static sitting with eyes closed, functional stability boundary

*For each app we considered the target population, risk factors specific to each population, design needs for each population, and solutions to meet these design needs*.

Compared to other fall risk apps, Steady, Steady-MS, and Steady-Wheels are some of the few apps that have been designed for specific clinical populations and evaluated for their validity, reliability, and usability. Greene et al. (26) used machine learning methods to develop an algorithm for predicting falls using mobile technology but did not test their app with older adult users. Mansson et al. (27, 28) developed an app to measure leg strength and balance and tested its usability with older adults but did not measure other fall risk factors. Taheri-Kharameh et al. (29) included educational recommendations in their mHealth app for users based on their fall risk. Each of these apps has its strengths, such as a robust, predictive algorithm, user-centered design, and fall management resources. When developing future fall risk apps, we recommend following the approach in Figure 1, building on the lessons we learned as described in Figure 2, and incorporating the strengths of other fall risk apps. Taking this approach can drive mHealth to a personalized falls prevention methodology.

Steady, Steady-MS, and Steady-Wheels offer a strong start in providing personalized fall risk screening for older adults, people with MS, and wheeled device users adults. To reduce falls and fall-related injuries in these groups, however, further work in the field of mobile technology is needed. First, within each fall risk app, more work is needed to develop a robust algorithm to measure fall risk. While previous studies have examined risk factors that are most predictive of falls, future work should include additional factors that can be measured with mobile technology. For instance, cognition was not measured in Steady, Steady-MS, or Steady-Wheels, and is a risk factor for falls in older adults, those with MS, and wheeled device users. Implementing cognitive testing should be a consideration for future iterations. Second, our collective work demonstrates that fall risk screening is valid, reliable, and usable across clinical populations, and a next step is to incorporate these apps into a fall prevention trial. Whether personalized fall risk screening leads to fewer falls remains unclear. Last, future work should bring fall risk apps into community settings. While each app was tested in home-based settings, they were tested under the supervision of research staff. Incorporating smartphone-based fall risk screening into virtual clinical visits or community interventions will determine whether they can be adapted into real-world settings.

In conclusion, mobile technology offers many advantages to increase access to personalized fall risk assessments. Our research group has taken an approach to develop fall risk apps for older adults, people with MS, and wheeled-device users. This development and real-world testing approach has demonstrated that when designed for the intended users, there is potential for mHealth fall risk screening in remote settings. Building upon our lessons learned and upon the strengths of previous groups can lead mHealth toward preventing falls across clinical populations.

## Author Contributions

KH drafted the main manuscript. MF drafted a section of the manuscript. KH and MF contributed to the studies included in the manuscript. JF developed the apps described. JS contributed to the conception and design of the studies included in the manuscript. All authors contributed to manuscript revision, read, and approved the submitted version.

## Funding

KH was funded by NIA T32 AG033534 and Wake Forest University Claude D. Pepper Older Americans Independence Center (P30 AG021332). JF was funded by Wells Fargo Faculty Scholar award and the Wake Forest University Claude D. Pepper Older Americans Independence Center (NIA P30-AG21332). JS was funded by NIH R21AG073892; NIH R21 AG064308-01; NIDILRR 90DPHF0010; NIDILRR 90REGE0006-01-00; National Multiple Sclerosis Society (MB-1807-31633; RG-1701-26862).

## Conflict of Interest

JS has ownership in Sosnoff Technologies, LLC received speaking fees from BrainWeek and consulting fees from Xavor, Inc. The remaining authors declare that the research was conducted in the absence of any commercial or financial relationships that could be construed as a potential conflict of interest.

## Publisher's Note

All claims expressed in this article are solely those of the authors and do not necessarily represent those of their affiliated organizations, or those of the publisher, the editors and the reviewers. Any product that may be evaluated in this article, or claim that may be made by its manufacturer, is not guaranteed or endorsed by the publisher.

## References

[1] Bergen G Stevens MR Burns ER. Falls and fall injuries among adults aged≥ 65 years—United States, 2014. Morbid Mortal Wkly Rep. (2016) 65:993–8. 10.15585/mmwr.mm6537a2 27656914

[2] Parkkari J Kannus P Palvanen M Natri A Vainio J Aho H. Majority of hip fractures occur as a result of a fall and impact on the greater trochanter of the femur: a prospective controlled hip fracture study with 206 consecutive patients. Calcified Tissue Int. (1999) 65:183–7. 10.1007/s002239900679 10441647

[3] Thompson HJ McCormick WC Kagan SH. Traumatic brain injury in older adults: epidemiology, outcomes, and future implications. J Am Geriatr Soc. (2006) 54:1590–5. 10.1111/j.1532-5415.2006.00894.x 17038079 PMC2367127

[4] Rubenstein LZ. Falls in older people: epidemiology, risk factors and strategies for prevention. Age Ageing. (2006) 35(Suppl. 2):ii37–41. 10.1093/ageing/afl084 16926202

[5] Scheffer AC Schuurmans MJ Van Dijk N Van Der Hooft T De Rooij SE. Fear of falling: measurement strategy, prevalence, risk factors and consequences among older persons. Age Ageing. (2008) 37:19–24. 10.1093/ageing/afm169 18194967

[6] Young WR Williams AM. How fear of falling can increase fall-risk in older adults: applying psychological theory to practical observations. Gait Post. (2015) 41:7–12. 10.1016/j.gaitpost.2014.09.006 25278464

[7] Guirguis-BlakeJMMichaelYLPerdueLACoppolaELBeilTL. Interventions to prevent falls in older adults: updated evidence report and systematic review for the US Preventive Services Task Force. JAMA. (2018) 319:1705–16. 10.1001/jama.2017.2196229710140

[8] Sherrington C Michaleff ZA Fairhall N Paul SS Tiedemann A Whitney J. Exercise to prevent falls in older adults: an updated systematic review and meta-analysis. Br J Sports Med. (2017) 51:1750–8. 10.1136/bjsports-2016-096547 27707740

[9] Tricco AC Thomas SM Veroniki AA Hamid JS Cogo E Strifler L. Comparisons of interventions for preventing falls in older adults: a systematic review and meta-analysis. JAMA. (2017) 318:1687–99. 10.1001/jama.2017.15006 29114830 PMC5818787

[10] Kramarow E CL Hedegaard H Warner M. Deaths from unintentional injury among adults aged 65 and over: United States, 2000–2013. In: Statistics NCfH, editor. NCHS Data Brief. Hyattsville, MD (2015). 25973998

[11] Stevens JA Phelan EA. Development of STEADI: a fall prevention resource for health care providers. Health Promot Pract. (2013) 14:706–14. 10.1177/1524839912463576 23159993 PMC4707651

[12] Ambrose AF Cruz L Paul G. Falls and fractures: a systematic approach to screening and prevention. Maturitas. (2015) 82:85–93. 10.1016/j.maturitas.2015.06.035 26255681

[13] Smith ML Stevens JA Ehrenreich H Wilson AD Schuster RJ Cherry CO. Healthcare providers' perceptions and self-reported fall prevention practices: findings from a large new york health system. Front Public Health. (2015) 3:17. 10.3389/fpubh.2015.00017 25964942 PMC4410324

[14] Mourcou Q Fleury A Franco C Klopcic F Vuillerme N. Performance evaluation of smartphone inertial sensors measurement for range of motion. Sensors. (2015) 15:23168–87. 10.3390/s150923168 26389900 PMC4610531

[15] Hsieh KL Fanning JT Rogers WA Wood TA Sosnoff JJ. A fall risk mhealth app for older adults: development and usability study. JMIR Aging. (2018) 1:e11569. 10.2196/11569 31518234 PMC6716481

[16] Bergen G Stevens MR Kakara R Burns ER. Understanding modifiable and unmodifiable older adult fall risk factors to create effective prevention strategies. Am J Lifestyle Med. (2019) 15:580–9. 10.1177/1559827619880529 34916876 PMC8669903

[17] Comber L Sosnoff JJ Galvin R Coote S. Postural control deficits in people with multiple sclerosis: a systematic review and meta-analysis. Gait Post. (2018) 61:445–52. 10.1016/j.gaitpost.2018.02.018 29486362

[18] Zhou J Habtemariam D Iloputaife I Lipsitz LA Manor B. The complexity of standing postural sway associates with future falls in community-dwelling older adults: the MOBILIZE Boston Study. Sci Rep. (2017) 7:1–8. 10.1038/s41598-017-03422-4 28592844 PMC5462759

[19] Rice LA Ousley C Sosnoff JJ. A systematic review of risk factors associated with accidental falls, outcome measures and interventions to manage fall risk in non-ambulatory adults. Disabil Rehabil. (2015) 37:1697–705. 10.3109/09638288.2014.976718 25354146

[20] LaPlante MP Kaye HS. Demographics and trends in wheeled mobility equipment use and accessibility in the community. Assist Technol. (2010) 22:3–17; quiz 19. 10.1080/10400430903501413 20402043

[21] Callis N. Falls prevention: identification of predictive fall risk factors. Appl Nurs Res. (2016) 29:53–8. 10.1016/j.apnr.2015.05.007 26856489

[22] Hsieh KL Roach KL Wajda DA Sosnoff JJ. Smartphone technology can measure postural stability and discriminate fall risk in older adults. Gait Post. (2019) 67:160–5. 10.1016/j.gaitpost.2018.10.005 30340129

[23] Hsieh KL Sosnoff JJ. Smartphone accelerometry to assess postural control in individuals with multiple sclerosis. Gait Post. (2021) 84:114–9. 10.1016/j.gaitpost.2020.11.011 33307327

[24] Hsieh K Fanning J Frechette M Sosnoff J. Usability of a fall risk mHealth app for people with multiple sclerosis: mixed methods study. JMIR Hum Fact. (2021) 8:e25604. 10.2196/25604 33749609 PMC8080269

[25] Hsieh KL Fanning JT Sosnoff JJ. A smartphone fall risk application is valid and reliable in older adults during real-world testing. Gerontechnology. (2019) 18:29–35. 10.4017/gt.2019.18.1.003.00

[26] Greene BR McManus K Ader LGM Caulfield B. Unsupervised assessment of balance and falls risk using a smartphone and machine learning. Sensors. (2021) 21:4770. 10.3390/s21144770 34300509 PMC8309936

[27] Mansson L Backman P Ohberg F Sandlund J Selling J Sandlund M. Evaluation of concurrent validity between a smartphone self-test prototype and clinical instruments for balance and leg strength. Sensors. (2021) 21. 10.3390/s21051765. [Epub ahead of print]. 33806379 PMC7961526

[28] Mansson L Wiklund M Öhberg F Danielsson K Sandlund M. Co-creation with older adults to improve user-experience of a smartphone self-test application to assess balance function. Int J Environ Res Public Health. (2020) 17. 10.3390/ijerph17113768. [Epub ahead of print]. 32466484 PMC7312460

[29] Taheri-Kharameh Z Malmgren Fänge A Ekvall Hansson E Bashirian S Heidarimoghadam R Poorolajal J. Development of a mobile application to screen and manage fall risks in older people. Disabil Rehabil Assist Technol. (2020) 1–6. 10.1080/17483107.2020.1785562. [Epub ahead of print]. 32608287

